# T cell-recruiting triplebody 19-3-19 mediates serial lysis of malignant B-lymphoid cells by a single T cell

**DOI:** 10.18632/oncotarget.2238

**Published:** 2014-07-23

**Authors:** Claudia C. Roskopf, Christian B. Schiller, Todd A. Braciak, Sebastian Kobold, Ingo A. Schubert, Georg H. Fey, Karl-Peter Hopfner, Fuat S. Oduncu

**Affiliations:** ^1^ Klinikum der Universität München, Medizinische Klinik und Poliklinik IV, Haematology/Oncology, Munich, Germany; ^2^ Ludwig-Maximilians-Universität München, Department of Biochemistry/Gene Center, Munich, Germany; ^3^ Klinikum der Universität München, Medizinische Klinik und Poliklinik IV, Division of Clinical Pharmacology, Munich, Germany; ^4^ Friedrich-Alexander-Universität Erlangen-Nürnberg, Department of Biology, Erlangen, Germany

**Keywords:** immunotherapy, triplebody, cytolytic T cells, antibody-dependent cellular cytotoxicity, leukemia

## Abstract

Triplebody 19-3-19, an antibody-derived protein, carries three single chain fragment variable domains in tandem in a single polypeptide chain. 19-3-19 binds CD19-bearing lymphoid cells via its two distal domains and primary T cells via its CD3-targeting central domain in an antigen-specific manner. Here, malignant B-lymphoid cell lines and primary cells from patients with B cell malignancies were used as targets in cytotoxicity tests with pre-stimulated allogeneic T cells as effectors. 19-3-19 mediated up to 95% specific lysis of CD19-positive tumor cells and, at picomolar EC_50_ doses, had similar cytolytic potency as the clinically successful agent Blinatumomab^TM^. 19-3-19 activated resting T cells from healthy unrelated donors and mediated specific lysis of both autologous and allogeneic CD19-positive cells. 19-3-19 led to the elimination of 70% of CD19-positive target cells even with resting T cells as effectors at an effector-to-target cell ratio of 1 : 10. The molecule is therefore capable of mediating serial lysis of target cells by a single T cell. These results highlight that central domains capable of engaging different immune effectors can be incorporated into the triplebody format to provide more individualized therapy tailored to a patient’s specific immune status.

## BACKGROUND

Therapeutic antibodies such as MabThera^®^, Herceptin^®^ and Avastin^®^ are potent and successful tools in the fight against cancer.[1] Recombinant proteins derived from conventional antibodies have been designed to increase tumor cell selectivity and deeper penetration into tumor tissues, and to exploit the patients' innate defense mechanisms against their disease.[2, 3] Several of the new antibody-derived agents - including immunoligands[4-6], diabodies, bispecific (bs) scFvs[7, 8], single chain triplebodies[9-12] and, most recently, a modular targeting system[13] - are based on single chain variable fragment (scFv) building blocks and lack an Fc region. While maintaining target specificity, the scFv-based agents are expected to reach deeper tissue penetration due to their reduced molecular mass.[14, 15] The lack of an Fc region is also thought to reduce undesired side effects, which are caused by binding to Fc receptors that are exposed on cells other than the desired cytolytic effectors.[2, 3]

Despite the lack of an Fc region, scFv-based agents have effective mechanisms of action. The anti-Her2/anti-Her3 bsscFv MM-111[16], for example, relies on simultaneous blockage of two different growth factor receptors and the cooperative inhibition of essential growth-promoting and anti-apoptotic downstream signals. Other mechanisms of action include the induction of apoptosis via surface receptors such as FasR (CD95)[17] and target-specific delivery of a toxin or radioisotope cargo by internalization (receptor-mediated endocytosis). Another option is the engagement of autologous effector cells, such as natural killer (NK)-cells and cytotoxic T lymphocytes (CTLs), for cytolysis.[2, 3]

NK cells can, for example, be recruited by the TandAb™ AFM-13 (CD30-CD16; Affimed) designed for the treatment of Hodgkin Lymphoma.[18] Examples for T cell recruitment via scFv-based agents are the bispecific T cell engagers (BiTEs; Micromet/Amgen). Antibody-derivatives in the BiTE-format coat their tumor cell targets and engage effector memory T cells independent of their MHC : peptide specificity via the CD3ε chain of the T cell receptor-CD3 (TCR/CD3) complex.[7] Coupling between T cell and tumor cell leads to the formation of a cytolytic synapse and degranulation of the T cell.[7, 19] Moreover, the engagement of effector memory T cells via BiTEs leads to their proliferation and thus to an expansion of the effector cell population that is available for tumor cell lysis during the course of the treatment.[20-22] The CD19-specific BiTE Blinatumomab^TM^ has shown impressive success in clinical studies with patients suffering from acute and chronic B cell malignancies.[20, 22-24] Other BiTEs targeting CD33 (CD33-CD3; AMG330)[25, 26], EpCAM (MT110; EpCAM-CD3)[27-29] and CEA (MT111; CEA-CD3)[30, 31], respectively, have produced promising results in recent pre-clinical studies and ongoing clinical trials.

However, despite their success, these bispecific agents still face limitations, which are difficult to overcome with this molecular format, including limitations in tumor cell specificity and “plasma retention time” (“plasma half-life”).[22, 23] The drawback in specificity is due to the fact, that the BiTE-format is monospecific and monovalent for the tumor cell, recognizing only one target antigen. While mono-targeting has been successful in several applications, it may not confer sufficient specificity for cancer cells and sufficient discrimination between cancer and healthy cells in many other cases. Given that spontaneously arising cancer cells share many antigens with healthy cells, and that many of the most promising targets, such as growth factor receptors (e.g. EGF-receptor) and cell adhesion molecules (e.g. EpCAM) are not tumor-specific, this situation will occur. Therefore, monospecific agents have often caused side effects that are difficult to manage. In addition, under therapy with monospecific agents, escape mutants of tumor cells can emerge, which have lost the surface expression of the corresponding target antigen and have become resistant to treatment.[32] Dual-targeting agents, which address two different target antigens on the same cancer cell, may be capable of overcoming some of these limitations. Also, most BiTEs have a molecular mass below 65 kDa and thus below the kidney excretion limit, which results in a plasma half-life in the range of approximately 1 hour.[33] This shortcoming can be addressed by agents with a higher molecular mass and by extending the half-life by PEGylation, by fusion to human serum albumin (HSA) domains carrying a binding site for the FcRn shuttling receptor, or antibodies and antibody fragments with specificity for HSA.[34]

To take advantage of these added capabilities offered by multispecific scFv-based agents, the molecular format of “single chain triplebodies” (“triplebodies”) has been developed by our group. A triplebody is composed of three scFvs connected via flexible (Gly_4_Ser)_4_ linkers, which results in a maximum computed distance of 20 nm between the two distal binding moieties[35] and a molecular mass of 85 to 95 kDa.[36] This mass exceeds the threshold for first-pass kidney excretion and consequently, triplebodies have a prolonged plasma half-life of approximately 4 hrs in mice. This corresponds to approx. 1 day in humans by allometric scaling.[9] Triplebodies can be designed to recruit different classes of effector cells via their central scFv, which is specific for an activating surface molecule such as CD16 on NK cells and macrophages[9, 10, 36], CD89 on polymorphonuclear granulocytes (PMNs)[37], or CD64 on macrophages and cytokine-stimulated PMNs.[3] The two distal binding moieties of the triplebody are capable of either monospecific bivalent targeting (example: 19-16-19)[9] or bispecific bivalent targeting (“dual-targeting”) of two different target antigens on the same cancer cell (examples: 123-16-33[36], 33-16-19[10] and HLA-DR-16-19[11]). Dual-targeting triplebodies have been shown to achieve preferential binding[4] and to mediate preferential lysis[38] of double-positive over single-positive target cells by cytolytic effectors in a mixed environment. They even permitted preferential effector-cell mediated lysis of the double-positive cells when the single-positive cells were present in an up to 20-fold numerical excess. For some target antigen combinations the dual-targeting approach may not be successful, because individual target affinities, antigen surface densities and the genetically and epigenetically determined susceptibility of the target cell to effector cell-mediated lysis need to cooperate for enhanced potency.[28] Nevertheless, dual-targeting triplebodies have been successful in preclinical studies for a number of different combinations of target antigens, and therefore this concept deserves further exploration.

The ability of triplebodies to recruit T cells as effectors is unaddressed so far and it is uncertain whether the fine structure of an immunological synapse mediated by a triplebody would be functional, as demonstrated for NK cells. In the present study a single chain triplebody was constructed and tested for the ability to recruit cytotoxic T cells (CTLs) as effectors for the lysis of CD19-positive leukemia cells. As we demonstrate in our current study, the new triplebody anti-CD19/anti-CD3/anti-CD19 (19-3-19) is capable of activating resting T cells and of mediating redirected and serial lysis of tumor cells, both of established B-ALL cell lines and of primary cells isolated from the peripheral blood of patients suffering from different types of B cell malignancies. The successful recruitment of effector T cells via 19-3-19 indicates that the triplebody platform can be used to recruit an even broader range of effector cells. The thus highlighted ability of the triplebody format to choose an optimally suited effector cell for therapy by adjusting the central trigger scFv based on a patient's specific cancer type and the availability of these effectors in the tumor tissue increases the range of therapeutic options and may add an additional layer to individualized cancer therapy.

## RESULTS

### Design and production of triplebody 19-3-19

**Figure 1:**
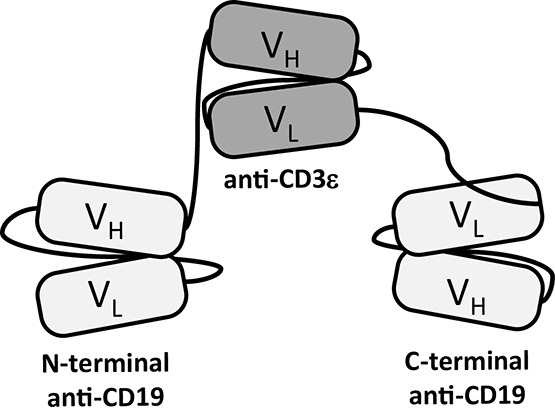
Domain architecture of T cell-recruiting triplebody 19-3-19 Triplebody 19-3-19 binds to the CD3ε chain of the TCR/CD3 complex via its central scFv and to two copies of CD19 on the surface of a malignant target cell via its two distal scFvs. The scFvs are connected by flexible (G_4_S)_4_-linkers (black lines), which gives the molecule a maximum computed span length (distance between the two distal binding sites) of 20 nm, when the flexible linkers are fully extended.

**Figure 2:**
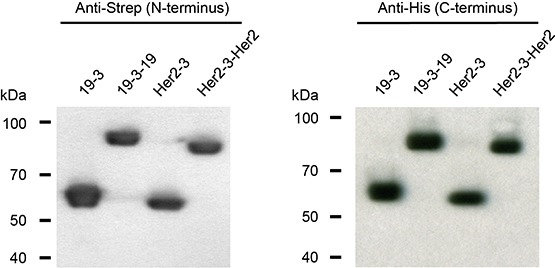
N- and C-termini of triplebody 19-3-19 and control proteins 19-3, Her2-3 and Her2-3-Her2 were intact The fusion proteins carried N-terminal Strep and C-terminal hexa-histidine tags. Western blot analysis with antibodies reacting with the Strep- and His-tags, respectively, revealed that the fusion proteins carried intact N- and C-termini and provided no indication for incomplete synthesis or proteolytic degradation.

Triplebody 19-3-19 carries a scFv domain derived from the murine hybridoma antibody OKT3 (directed against human CD3ε) in the central position, and CD19-specific scFv domains, targeting the B-lineage antigen CD19, derived from hybridoma 4G7 as previously described (Fig. 1). [9] In this fusion protein with an N-terminal Strep-tag and a C-terminal His-tag, the CD19-specific scFv components were humanized by CDR-grafting.[39] Triplebody 19-3-19 was expressed both in adherent HEK 293T cells and in suspension-adapted HEK 293F cells with yields between 0.9 and 5.1 mg protein/L culture supernatant in different experiments (Table 1). HEK 293F cells permitted cultivation in serum-free medium and efficient purification via Ni-NTA affinity chromatography. The N- and C-termini were intact and no degradation products were detected (Fig. 2). In addition, two new agents in the Bispecific T cell Engager (BiTE)-format were produced as controls. One carried our humanized CD19-specific scFv domain and was designated 19-3 (“Blinatumomab™-look-alike”), the other carried scFvs with specificity for Her2/NEU and CD3ε and was designated Her2-3. Finally, a control triplebody targeting Her2/NEU (designated Her2-3-Her2) was also produced. Expression of Her2-3-Her2 and Her2-3 was efficient with yields of 8.5 and 9.2 mg/L of culture supernatant, respectively (Table 1).

**Table 1:** Molecular masses and yields of T cell-engaging triplebodies and bispecific scFvs Proteins were expressed and purified as described in the Methods section. Buffer conditions were chosen to minimize protein aggregation. Proteins were concentrated to 150 - 300 ng/μL in the storage buffer by centrifugation, using centrifugal filters with a molecular weight cut-off (MWCO) of 50 kDa for the triplebodies and 10 kDa for bispecific scFvs. Theoretical molecular masses were computed from the known amino acid sequence composition of the molecules.

Protein	Computed Mass [kDa]	Yield [mg/L] (293F cells)	Storage Buffer
anti[CD19-CD3]	60.7	0.9 – 4.8	20mM L-Histidin 300mM NaCl 100mM D-Trehalose 5mM EDTA 10% Glycerol pH 6.5
anti[CD19-CD3-CD19]	89.9	0.9 – 5.1	
anti[Her2-CD3]	59.5	1.8 – 9.2	
anti[Her2-CD3-Her2]	87.2	6.0 – 8.5	

### Triplebody 19-3-19 binds specifically both to its target antigens and the trigger protein

**Figure 3:**
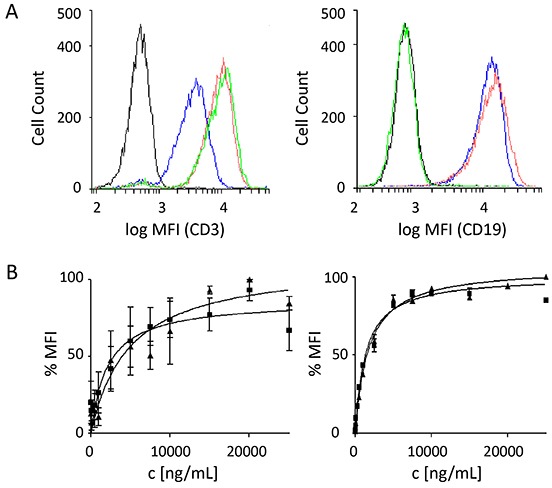
Binding specificities of the scFv components of triplebody 19-3-19 Target specificity of the 19-3 BiTE protein and triplebody 19-3-19 was examined by flow cytometry as described.[53] Molecules bound to the surface of single-positive target cells were detected with a secondary anti-His mAb and a Phycoerythrin (PE)-conjugated tertiary goat-anti-mouse IgG mAb. (**A**) Shift in mean fluorescence intensity (MFI) produced by binding to primary T cells (left), and Nalm-6 cells (right) at a saturating concentration of 15 μg/mL of either the BiTE or the triplebody. Black: isotype control; blue: triplebody 19-3-19; red: 19-3 BiTE; green: control triplebody Her2-3-Her2. MFIs are given as logarithms to the base of 10. (**B**) Determination of equilibrium dissociation constants K_D_ of 19-3 and the triplebody 19-3-19 for CD3 on primary T cells (n = 4), and for CD19 on SEM cells (n = 7). Error bars indicate standard error of the mean (SEM). The dissociation constants for CD3 were 34.7 ± 14 nM and 53.3 ± 19 nM for the BiTE and the triplebody, respectively. The dissociation constants for CD19 were 28.4 ± 1 nM for 19-3 and 14.7 ± 2 nM for triplebody 19-3-19, where the latter value refers to the overall (bivalent) avidity of the entire molecule, not to the monovalent affinity of the individual CD19-specific scFvs.

Both the triplebody 19-3-19 and the BiTE 19-3 bound to primary human T cells isolated from *ex vivo* expanded mononuclear cells (Fig. 3A; left), as well as to CD19-positive Nalm-6 cells (a pre-B ALL-derived cell line; Fig. 3A, right), but it did not bind to antigen-negative HEK 293F cells (data not shown). The Her2-3-Her2 specificity control bound to T cells via the trigger CD3ε, but not to Her2- and CD3ε-negative Nalm-6 cells. At the saturating concentration of 15 μg/mL both the control triplebody Her2-3-Her2 and the 19-3 BiTE showed stronger binding to T cells than triplebody 19-3-19, as evidenced by a stronger shift in the mean fluorescence intensity (MFI) of the cell-bound fusion proteins detected by cytofluorimetry (Fig. 3A, left panel). Thus the binding capacity of the CD3ε-specific scFv domain was affected by its molecular context within a given fusion protein. The difference in binding strength was also reflected in the equilibrium dissociation constants (K_D_ values) of 19-3-19 and 19-3 for CD3ε exposed on primary T cells. The triplebody bound less strongly with an affinity of 53.3 ± 19 nM compared to 34.7 ± 14 nM for the BiTE 19-3 (Fig. 3B, left panel), but the difference was not significant. The overall avidity of the triplebody for CD19 on the surface of SEM (pro-B ALL) cells was 14.7 ± 2 nM. Thus, the binding-strength of the triplebody for CD19 was approximately two-fold greater than the monovalent affinity of the CD19-specific scFv-domain carried in the control 19-3 with a K_D_ value of 28.4 ± 1 nM (Fig. 3B, right panel). These numerical values indicate that the two CD19-specific scFv domains of triplebody 19-3-19 contributed to the overall avidity of this protein in an additive rather than a synergistic manner, which was previously reported for the triplebody 19-16-19.[9] This observation suggests that the detailed spatial arrangement assumed by the two CD19-specific scFvs in a triplebody, which mediate the association with a target cell, is different between an NK- and a T cell-recruiting agent. The increase in avidity for CD19 on living cells observed for the triplebody relative to the BiTE is also evidence that both CD19-binding sites of the triplebody can simultaneously bind one copy each of CD19 on the same target cell.

### Triplebody 19-3-19 mediates specific target cell lysis in combination with effector T cells

**Figure 4:**
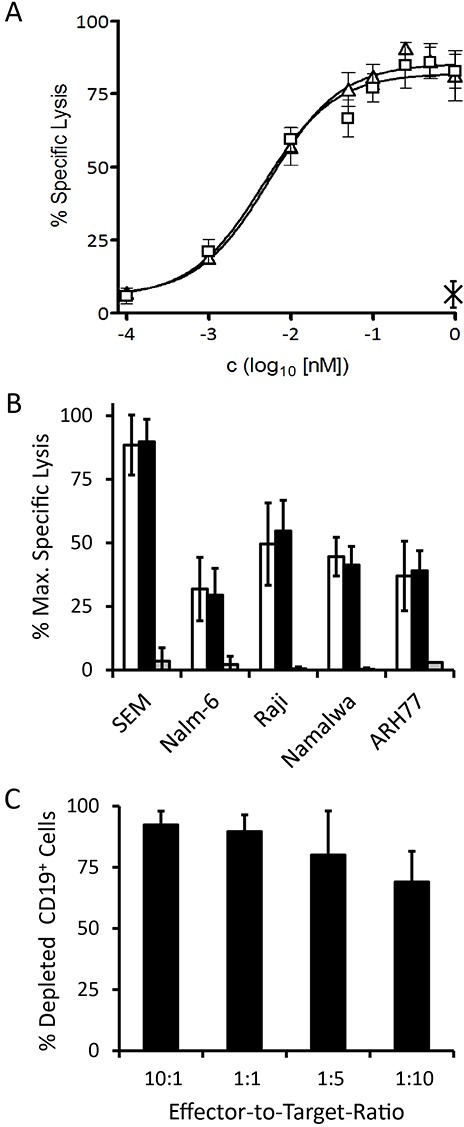
Specific redirected lysis of malignant target cell lines mediated by the 19-3 BiTE and triplebody 19-3-19 in conjunction with effector T cells Standard Calcein Release Assays with an E : T ratio of 10 : 1 (MNCs : target cells) and a duration of 3 hrs were performed unless indicated otherwise. Error bars indicate SEM. (**A**) Dose-Response-Curve for SEM cells (triangles: triplebody 19-3-19; squares: 19-3 BiTE; cross: specificity control Her2-3-Her2; n=4). (**B**) Maximum specific lysis of several CD19-positive malignant B-lymphoid cell lines (n=4 each) induced by 19-3 (white bars), triplebody 19-3-19 (black bars) and specificity control Her2-3-Her2 (grey bars), respectively. (**C**) Serial lysis of unlabeled SEM target cells mediated by triplebody 19-3-19 at different E : T ratios. The reaction mixture (purified T cells and SEM target cells in RPMI1640 GlutaMAX medium containing 10% FCS) was incubated with or without 1nM triplebody overnight (approx. 15 hrs) (n = 3). The total number of viable cells in each reaction was determined and the fraction of CD19-positive target cells was established by FACS analysis.

To investigate whether the formation of a cytolytically productive synapse between an effector T cell and its tumor cell target can be mediated by triplebody 19-3-19, redirected lysis (RDL) assays were performed. For this purpose, a panel of CD19-positive leukemia- and lymphoma-derived cell lines representing different types of B cell-malignancies were used as targets with a T cell: target cell ratio of 6: 1. Triplebody 19-3-19 or control proteins 19-3 and Her2-3-Her2 were added at different concentrations and after a 3 hr reaction time target cell death was measured by Calcein release.[11, 38] 19-3-19 and 19-3 produced significant specific lysis of CD19-positive target cells in a dose-dependent manner (Fig. 4a). However, even at the highest concentration of 10 nM, the specificity control triplebody Her2-3-Her2 did not produce any significant specific lysis (3.5 ± 5% background). This result is in accordance with an earlier report[40] describing that the sole binding of a BiTE protein to CD3ε on the T cell in the absence of binding to the target cell was not sufficient to induce bystander lysis of target cells by the T cells.

Lysis of SEM cells occurred with a sigmoidal dose-response with a maximum specific lysis of 89.8 ± 9% after 3 hrs (mean of 4 experiments) and an EC50-value of 5.5 pM (95% CI: 3-9 pM). Dose-responses were also recorded for Nalm-6 (pre-B ALL), Raji (Burkitt lymphoma), Namalwa (Burkitt lymphoma) and ARH77 (plasma cell leukemia) cells and EC50-values were in the low picomolar range (Table 2). The specificity control triplebody Her2-3-Her2 did not induce significant lysis of any of the target cell lines (Fig. 4B). Different surface antigen densities of CD19 on these cell lines are an explanation for the difference in lysis measured, because a loose correlation was observed between the copy number of CD19 molecules per cell and the degree of maximum specific lysis (Fig. 4B and Table 2). Copy numbers per cell (given in the parentheses) were measured by calibrated cytofluorimetry and gave rise to the following ranking: SEM (30,000 ± 8,000) > Raji (23,500 ± 19,000) > Nalm-6 (17,500 ± 7,000) > Namalwa (7,000 ± 4,000) > ARH77 (1,500 ± 2,000). Numbers of CD19 copies per cell were weakly correlated with the degree of maximum specific lysis achieved for these cell lines (Table 2) with the exception of Nalm-6 cells. Nalm-6 cells carried intermediate numbers of CD19 copies per cell but responded poorly to lysis mediated by the triplebody plus T cells. Finally, no statistically significant difference between the degree of maximum lysis of these cell lines reached by T cells in combination with either the triplebody 19-3-19 or the BiTE 19-3 was observed (p-values > 0.05; Table 2). Thus, both agents mediated comparable maximum lysis of different types of malignant B-lymphoid cells.

**Table 2:** Maximum lysis and EC50 values of 19-3 bispecific scFv and triplebody 19-3-19 for different malignant B-lymphoid cell lines

Cell Line		Maximum Specific Lysis [%]			EC50 [pM] (95% CI [pM])			n
		19-3	19-3-19	p-value	19-3	19-3-19	p-value	
pro-B ALL	**SEM**	88.5 ± 12	89.8 ± 9	0.33	4.5 (2 - 10)	5.5 (3 - 9)	0.43	4
	**Nalm-6**	31.9 ± 12	29.5 ± 11	0.08	12.7 (5 - 30)	22.3 (16 - 31)	0.21	4
Burkitt lymphoma	**Raji**	49.6 ± 16	54.7 ± 12	0.1	1.8 (0.5 - 7)	47.4 (11 - 201)	0.053	4
	**Namalwa**	44.6 ± 8	41.3 ± 7	0.12	6.2 (32 - 12)	19.9 (9 - 43)	0.071	4
plasma cell leukemia	**ARH77**	37.0 ± 14	39.0 ± 8	0.34	5.6 (0.3 - 95)	189.6 (54 - 664)	0.003	4

### Triplebody 19-3-19 induces serial tumor cell lysis

Cytolytic T cells alone and T cells recruited by Blinatumomab^TM^ are capable of serial target cell lysis.[19] This is a valuable property for therapeutic efficacy, and therefore we sought to determine, whether T cells recruited by 19-3-19 were also capable of serial lysis. Redirected lysis experiments were performed for an extended reaction period of 15 hrs with a constant saturating concentration of 19-3-19 (1 nM), constant numbers of target cells, but decreasing effector-to-target cell ratios over the range from 10 : 1 to 1 : 10 (Fig. 4C). Even at an effector : target cell ratio of 1 : 10, 69 ± 13% of the CD19-positive target cells were lysed, which provides clear evidence for serial lysis by T cells mediated by the triplebody.

### Activation of resting T cells via synapse-formation mediated by 19-3-19

Under physiological equilibrium conditions T cells in human blood are not activated unless an immune response is raised to a pathogen. Consequently, a therapeutic agent based on T cell-engagement may also require the activation of resting T cells to develop maximum efficacy. The BiTE Blinatumomab^TM^ has already been shown to cause the activation of memory T cells, which (differing from naive T cells) do not require a second activation signal, and to trigger their proliferation.[20-22] To investigate whether triplebody 19-3-19 is capable of activating resting T cells when it connects the target and effector cell via the CD3ε-specific scFv domain, a long-term (72 hr) RDL assay was performed using non-stimulated PBMCs from healthy unrelated donors as a source of effector cells and SEM cells as targets at an E : T ratio of 10 : 1 (PBMCs : targets).

The population of PBMCs from healthy donors includes both CD19- and CD3-positive cells and therefore, the frequencies of these subsets within the PBMC populations were measured for each donor prior to their use in RDL assays. For the panel of donors tested, the CD19-positive subset ranged from 0.8 to 12.6% and the T cell subset from 69.3 to 74.5% of all PBMCs, respectively.

**Figure 5:**
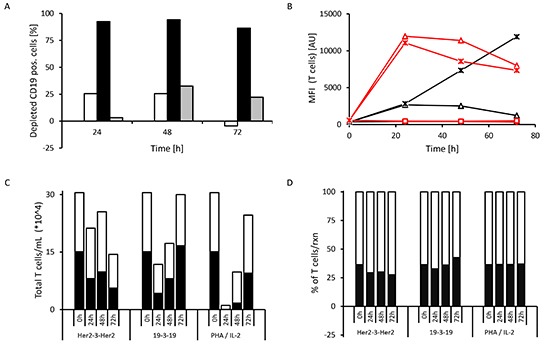
Example of T cell activation induced by triplebody 19-3-19 Long-term (72 hrs) redirected lysis assay with an E : T ratio of 10 : 1 non-stimulated PBMCs : SEM target cells and 1 nM triplebody or 2% PHA/100 U/mL IL-2 (pos. control) were performed and the expression of activation markers by the effector T cells was assessed at 0, 24, 48 and 72 hrs (n=4). Representative data from one 28-yr old, healthy male donor are shown. The CD3-positive cells comprised 70.4% of the donor's PBMC fraction and the CD19-positive cells of the donor comprised 4.5% of his PBMCs. At time t_0_ the overall content of CD19-positive cells in the reaction mixture (PBMCs + SEMs) was 14.6%. (**A**) Depletion of CD19-positive target cells over time (white bars = triplebody Her2-3-Her2; black bars = triplebody 19-3-19; grey bars = 2% PHA/100 U/mL IL-2). (**B**) Time course of expression of early T cell activation marker CD69 (red) and late activation marker CD25 (black) on the cell surface. The increase in activation marker expression coincided with elevated IFN-γ concentrations in the supernatant (data not shown). Diamonds: no treatment; squares: treatment with triplebody Her2-3-Her2; triangles: treated with triplebody 19-3-19; crosses: treated with 2% PHA/100 U/mL of IL-2. (**C**) Absolute number of T cells (CD3^+^ cells) and memory T cells (CD3^+^ CD45RO^+^, dark area) in the reaction mixture. **(D)** Fraction of memory T cells (CD3^+^ CD45RO^+^, dark area) in the total CD3-positive cellular compartment, which was set to 100%.

At the start of these experiments (time t_0_) the CD19-positive cellular subset accounted for 11.6 to 19.6% of all cells (PBMCs plus target SEM cells) in the reaction mixture. The decreasing fraction of CD19-positive cells, the number of memory T cells (CD3^+^ CD45RO^+^), the expression levels of the early T cell activation marker CD69 (trans-membrane C-type lectin) and of the late activation marker CD25 (α-subunit of the IL-2 receptor) on CD3-positive cells were then assessed every 24 hrs (Fig. 5).

T cell activation was induced in all donor samples tested, but with extensive inter-donor variability (data not shown). The T cell activation profile from one particular 28-yr old male donor is shown in Fig. 5. After 24 hrs 92.5% (values ranging from 86.0 to 99.4% lysis for different donors) of CD19-positive target cells were depleted (Fig. 5A). Maximum depletion of targeted cancer cells was generally achieved after 48 hrs (94.2 to 99.6% lysis). The control triplebody Her2-3-Her2 did not induce T cell activation as determined by activation marker expression (Fig. 5B) and Interferon-γ release (data not shown).

In all donor samples the addition of 19-3-19 or PHA/IL-2 (positive control) to the reaction mixture caused a strong increase of the early activation marker CD69 on the surface of the CD3-positive cells (Fig. 5B, red) within the first 24 hrs. At later time points the CD69 levels dropped progressively for the rest of the time course. Surface expression of CD25 was also induced, but less intensely and slower than CD69 and also varied greatly among the different donor samples. CD25 expression levels, induced by the triplebody, peaked between 24 to 48 hrs and remained almost constant for the later time points (Figure 5B, black). In contrast, addition of the Her2-3-Her2 control triplebody caused no changes in CD69 or CD25 expression in the CD3-positive T cells. Addition of the triplebody 19-3-19 alone to the effector cells in the absence of CD19-positive target cells did not have any effect on the T cell population (data not shown). Therefore, the triplebody only caused activation of the effector T cells, when it physically connected the effector and the target cells in an antigen-specific manner and engaged them to build a productive synapse.

In the redirected lysis (RDL) experiments with triplebody 19-3-19 or PHA/IL-2 the overall numbers of living T cells dropped during the first one to two days, but rose again and reached the numbers present in the starting population after 72 hrs (shown for the 28-yr old male donor sample in Fig. 5C). The subset of the CD3-positive population defined as memory T cells (CD3^+^ CD45RO^+^) also followed the drop and increase over time (Fig. 5D). We conclude that the T cell-recruiting triplebody can engage and activate memory T cells and induce their proliferation.

### Triplebody 19-3-19 mediates efficient lysis of CD19-positive malignant cells in primary patient samples

To determine the effect of an *ex vivo* treatment of patient cells with triplebody 19-3-19 plus effector cells, primary patient samples were used as malignant targets. PBMC fractions from five patients with different types of B cell malignancies were isolated by density gradient centrifugation and then employed as targets in our Calcein release RDL assays. The first patient suffered from an unusual case of tri-phenotypic acute leukemia (expressing B- and T-lymphoid plus myeloid lineage markers) and donated blood at diagnosis. Whole blood samples were also collected at diagnosis from an immunocytoma patient and two B-CLL patients. Finally, a blood sample from a B-CLL patient, who had relapsed 4 years after a complete remission and who had received 6 courses of combination therapy using MabThera^®^ plus fludarabine and cyclophosphamide, was collected. This patient still displayed low expression of approximately 1,800 copies/cell of CD20 on its surface (Table 3, bottom). Patient characteristics are summarized in table 3.

**Table 3:** Synopsis of patient data Patient characteristics (top), target and effector cell content (center) and specific antibody binding capacity for CD3, CD19, CD20 and CD33 of malignant cells in the peripheral blood as determined with the QIFIKIT (Dako) (bottom).

		Patient 1 (green)	Patient 2 (red)	Patient 3 (blue)	Patient 4 (yellow)	Patient 5 (purple)
Gender		Male	Male	Male	Female	Male
Age		19 years	86 years	78 years	67 years	52 years
Diagnosis		Mixed phenotype acute leukemia (MPAL (NOS))	CD19-positive NHL with leukemic progression	B-CLL	Relapsed B-CLL	B-CLL
Blast titer		93.4% blasts in BM at diagnosis	58% of lymphocytes in the PB are CD19-positive	29,000 B-CLL cells/μL	65% lymphocytes in the PB (9% atypical)	42% lymphocytes in the PB
Case history		newly diagnosed	newly diagnosed	newly diagnosed	4 years prior: 6x MabThera^®^ plus fludarabine and cyclophosphamide > CR	newly diagnosed
% CD19+ in PBMCs		94.8	21	89.8	68.8	73.2
% CD3+ in PBMCs		0.8	56.1	6.8	16.6	8.6
Specific antibody binding capacity (SABC) of tumour cells in the peripheral blood	CD3	211	n.d.	n.d.	n.d.	n.d.
	CD19	8,400 ± 2,800	14,600 ± 7,700	9,600 ± 500	6,500 ± 1,700	7,600 ± 1,900
	CD20	0	19,400 ± 2,200	4,000 ± 100	1,800 ± 300	3,900 ± 1,200
	CD33	317	n.d.	n.d.	n.d.	n.d.

Dose-responses for each patient sample were determined, using either our 19-3 BiTE, the triplebody 19-3-19, or the therapeutic CD20 antibody MabThera^®^ and were assessed with standard 3 hr Calcein release-assays using allogeneic, *ex vivo* expanded MNC effector cells (expanded in the presence of IL-2) from an unrelated healthy donor. The E : T ratio (MNCs : targets) was 10 : 1, which corresponded to an actual T cell : target ratio of 5 : 1 and an NK cell : target ratio of 2 : 1.

**Figure 6:**
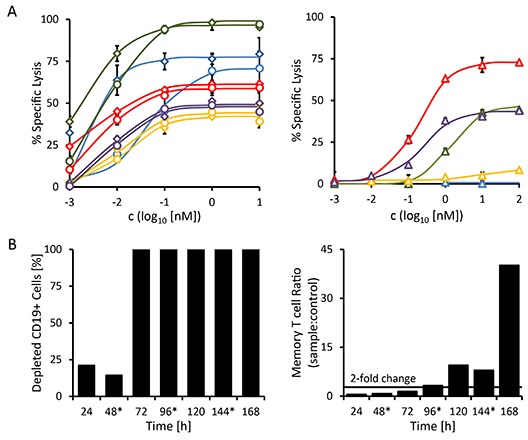
Redirected lysis of CD19-positive malignant cells from primary patient material mediated by triplebody 19-3-19 The ability of triplebody 19-3-19 to mediate the lysis of malignant cells isolated from the peripheral blood of patients with different B cell malignancies (see Table 3) via allogeneic and autologous effector T cells was assessed in redirected lysis assays. Samples were assayed in triplicate. Error bars indicate intra-sample variation. (**A**) Specific lysis of malignant cells via allogeneic pre-stimulated effector T cells mediated at different concentrations of the 19-3 BiTE (diamonds) and triplebody 19-3-19 (circles) or the therapeutic antibody MabThera^®^ (triangles) in a 3 hr assay, respectively. Patient 1 (MPAL (NOS)): blue; patient 2 (NHL): red; patient 3 (B-CLL): green; patient 4 (relapsed B-CLL): yellow; and patient 5 (B-CLL): purple. (**B**) Triplebody 19-3-19-induced depletion of CD19-positive cells by autologous effector T cells and expansion of memory T cells (CD3^+^ CD45RO^+^) in the PBMC fraction isolated from patient 2 (NHL) in a 7 d assay. 1 nM of fresh Triplebody 19-3-19 was added every 48 hrs (indicated by asterisks). A similar assay was performed with samples from patients 1 (MPAL (NOS)) and 3 (B-CLL) (data not shown), but no response was observed, possibly due to the relatively short observation time, T cell attenuation or too low initial numbers of effector T cells.

The maximum cytolytic response of the three CD20-positive B-CLL patient samples (patients 3, 4 and 5) to both CD19-targeting antibody-derivatives was more pronounced than the response to the standard-of-care reagent MabThera^®^ (Fig. 6A). The malignant cells of these three patients had higher surface densities of CD19 than of CD20 (Table 3, bottom), which paralleled the higher maximum specific lysis mediated by 19-3 and 19-3-19 than by MabThera^®^. The sample from the immunocytoma patient (patient 2) responded more strongly to treatment with MabThera^®^ than to treatment with either 19-3-19 or 19-3 (Fig. 6A). This coincides with a greater surface density of CD20 than CD19 on the malignant target cells from this patient (Table 3, bottom). The mixed phenotype acute leukemia (not otherwise specified) (MPAL(NOS)) patient sample (patient 1) was CD20-negative and consequently failed to display any response to treatment with MabThera^®^, but it did respond to treatment with both the 19-3 and 19-3-19 reagents (Fig. 6A). The response profile of this MPAL patient is more typical for patients with acute B-lymphoblastic leukemia often found in children and young adults, where the malignant blasts have a phenotype resembling early stages of B cell differentiation. Blasts from B cell precursor leukemias (BCP-ALLs) of infants, children and young adults often fail to express CD20, and thus these patients are more likely to benefit from treatment with a CD19-directed rather than a CD20-specific agent.[41, 42]

Triplebody 19-3-19 and our 19-3 BiTE reagent were effective at far lower concentrations than MabThera^®^. Although inter-patient variation was substantial, the EC50-values for 19-3 and 19-3-19 generally were 30- to 925-fold lower than those determined for MabThera^®^ for the panel of patient samples analyzed here (Table 4). No statistically significant differences between the BiTE reagent and the triplebody were observed with regard to the degree of maximum lysis and the EC50 values. Interestingly, maximum lysis achieved with the triplebody was marginally lower than the extent reached with 19-3. Maximum specific lysis and EC50 values are summarized in Table 4.

**Table 4:** Synopsis of maximum lysis and EC50 values for patient samples

	Maximum Specific Lysis [%]				EC50 [pM] (95% CI [pM])		
	19-3	19-3-19	MabThera^®^	19-3	19-3-19	MabThera^®^
**Patient 1**	79.3	70.8	n.d.	1.2 (0.3 - 5)	48.1 (9 - 270)	n.d.
**Patient 2**	61.5	59.2	72.8	6.1 (4 - 10)	4.8 (3 - 9)	185.7 (135 - 255)
**Patient 3**	98	100	44.5	2.8 (1 - 8)	6.4 (4 - 11)	1,300 (426 – 4,224)
**Patient 4**	49.4	44.7	8.4	10.7 (1 - 177)	13.9 (2 - 84)	9,900 (0.1 - 568 nM)
**Patient 5**	51.3	48.8	43.9	6.7 (1 - 36)	7.1 (2 - 29)	247.5 (89 - 688)

### Triplebody 19-3-19 induces expansion of memory T cells in a sample from an NHL patient

In patients with malignant hematopoietic diseases healthy immune effector cells are often displaced and suppressed. However, sufficient numbers of functioning effector cells are needed for a successful therapy with an agent critically relying on the recruitment of cytolytic effectors. By inducing the proliferation of memory T cells, the BiTE^®^ agents Blinatumomab^TM^ and AMG330 have caused an amplification of available effector cells.[20-22, 26] As the triplebody 19-3-19 also employs an OKT3-derived scFv-domain to trigger its effector cells, we investigated whether exposure to this triplebody was also capable of stimulating the proliferation of memory T cells in a patient sample. To this effect, the triplebody was added in a 1 nM saturating concentration to PBMCs isolated from an immunocytoma patient (patient 2) every 48 hrs for 7 days. At timepoint t_0_, the sample contained 21% CD19-positive cells and 56.1% CD3-positive cells (Table 3). Approximately half of the CD3-positive cells (53.3%) expressed the memory T cell marker CD45RO on their surface. In this particular patient sample, the T cell population displayed the same initial drop in numbers during the first 24 hrs that was also observed during the activation of T cells in the healthy donor samples reported above (Fig. 5). However, after 48 hrs, the T cells started to expand and the numbers of memory T cells had more than doubled after 96 hrs in comparison to the control reaction without added triplebody. On day 7, the numbers of memory T cells were increased by more than 42-fold in the triplebody-treated sample (Fig. 6B, right). The overall T cell population expanded 7-fold during the 7 d period of observation despite the absence of detectable numbers of CD19-positive target cells from 72 hrs on (Fig. 6B, left).

## DISCUSSION

In the present study, the prototype T cell-recruiting triplebody 19-3-19 was constructed and shown to engage pre-activated as well as non-stimulated T cells efficiently for the redirected and serial lysis of malignant CD19-positive target cells. Moreover, 19-3-19 led to the activation and induced the proliferation of allogeneic and autologous effector T cells. The key new result of the present study is the observation that fusion proteins in the molecular format of single chain triplebodies are also suited for the engagement of cytolytic T cells (CTLs) via CD3ε to eliminate antigen-positive cancer cell targets.

The concept of an “individualized therapy” usually refers to the selection of a specific therapeutic target based on a patient's individual genotypic and phenotypic characteristics, as determined by molecular diagnostics. However, another important aspect of personalized medicine, especially when planning to exploit or boost the patient's own immune system, is the size and activity of a suitable population of immune effector cells available at the cancer site. As a patient's immune status varies with the type and stage of the disease, different effector cell populations may be best suited in different situations. NK cells, for example, recover more rapidly than T and B lymphocytes after chemotherapy, and they require little activation time. They are present in the human circulation in a pre-activated state and are instantaneously ready for antibody-mediated cytolysis. Thus NK cells may be a suitable choice of effector cells to combat minimal residual disease after an induction chemotherapy.[3] CD8^+^ T cells, however, are frequently present in cancer tissues in abundance, have a greater intrinsic cytotoxic potential than NK cells, and are capable of more prolonged serial lysis.[43, 44] CTLs are therefore a particularly desirable class of cytolytic effectors for cancer therapy.

Ideally, for a fully individualized approach and to avoid successful immune evasion by the tumor cells, both the therapeutic target and the effector population should be chosen corresponding to the patient's individual disease properties. The molecular format of triplebodies may help to reach this goal, because triplebodies were found suited in this study to recruit not only NK cells but also T cells as cytolytic effectors.

Both the protein 19-3 in the BiTE-format and the newly constructed triplebody 19-3-19 were produced with similar expression yields and had similar cytolytic potential. Triplebody 19-3-19 led to efficient *in vitro* lysis of CD19-positive targets from both established malignant B-lymphoid cell lines and primary patient material at picomolar concentrations. Cell surface density of target antigens was a major, but not the only important determinant of cytolytic efficacy of the agent. A CD19-positive cell line with high antigen surface expression (SEM) showed a greater degree of maximum lysis in a standard 3 hr reaction interval in our redirected lysis experiments than cell lines with lower surface expression of the target antigen (Raji, Namalwa and ARH77).

The difference between maximum lysis of SEM cells (89.8%) and Raji cells (54.7%) was significant, even though CD19 copy numbers per cell differed by only 6,500 copies between these two cell lines. However, the determination of antigen copy numbers per cell by calibrated cytofluorimetry does not take the actual cell size into account and therefore produces copy numbers per cell, but not antigen density values. We have observed that Raji cells (Burkitt's lymphoma-derived) are much larger than SEM cells (pro-B ALL-derived), and therefore, the antigen density per surface unit may be lower for Raji cells than for SEMs. However, this argumentation does not fully explain the low specific lysis achieved for Nalm-6 cells. Nalm-6 cells have a relatively small cell volume that is comparable to SEMs and carried 17,500 copies of CD19/cell, but resulted in only 31.9 and 29.5% specific lysis mediated by 19-3 and 19-3-19, respectively, which were the lowest values observed. Additional parameters other than antigen surface density appear to play important roles in determining the sensitivity of a target cell to T cell-mediated lysis, probably linked to the specific oncogenic genomic and epigenetic alterations of the particular target and their tumor-type specific susceptibilities to apoptosis.

The triplebody 19-3-19 induced a similar T cell response profile as Blinatumomab^TM^. Activation of the T cell by this triplebody only occurred when the T cells were connected to the target cells by the triplebody in an antigen-specific manner, but was independent of specific MHC : peptide recognition. Furthermore, memory T cells rather than naïve T cells were engaged and induced to proliferate. Whether the triplebody also affects the activation state of naïve T cells, i.e. whether naïve T cells remain resting or are forced into anergy due to the interaction of 19-3-19 with CD3 in the absence of any “second signal” (co-stimulus), remains to be determined.

Interestingly, the CD3ε-specific scFv of 19-3-19 bound less efficiently to primary T cells than when this domain was embedded in our 19-3 BiTE protein or the Her2-3-Her2 control protein, when each was used at saturating concentrations. Together with the lower affinity of 19-3-19 for CD3ε in comparison to 19-3, these observations indicate that CD3ε on the surface of the T cells is less available for binding by the triplebody 19-3-19 than by triplebody Her2-3-Her2 and by the 19-3 protein. We cannot yet offer a definitive explanation for this observation, but the precise conformation of the CD3ε-specific scFv is likely different, when it is flanked at both sides by CD19-specific scFvs or Her2-specific scFvs, or only at one side by a single CD19-specific scFv. One possibility may be partial sterical hindrance of the CD3ε-specific scFv by the two distal CD19 scFvs, which may impact affinity.

A sensitivity of CD3ε-specific scFvs to their precise molecular surroundings has been previously reported by other authors. In the hands of M. Arndt and colleagues the CD3ε-specific scFv required optimization for every different BiTE construct, which led to the development of a new modular targeting system by these authors.[13] Further development of previously reported trispecific T cell-engaging antibody-derivatives, which employed an N-terminal tumor-associated antigen (TAA)-specific scFv, a central CD3ε-specific scFv or V_H_ domain, and a C-terminal CD28-specific V_H_ domain in one polypeptide chain,[45, 46] was discontinued. The question, whether the CD3ε-specific scFv domain in the central position of these fusion proteins was stable and functional, remained unanswered.

The value of triplebodies recruiting effector T cells can most likely be further increased by taking advantage of the unique capability of triplebodies for “dual-targeting”. Dual-targeting has been reported to enhance the selectivity of triplebodies recruiting NK cells as cytolytic effectors,[38] but it needs to be determined whether a preferential lysis of antigen double-positive cells over simultaneously present antigen single-positive cells can also be achieved by dual-targeting triplebodies, which recruit T cells as effectors. This may not be a foregone conclusion, because results recently reported by Harms and colleagues about the cross-arm binding efficiencies of monoclonal antibodies and different multispecific antibody-derived formats[47] suggest, that not every target combination leads to an improved antibody-activity in spite of the higher combined target antigen density. This may be explained in part by the “protein island”-model of the cell membrane, which is currently one of the most potent models for the surface architecture of human leukocytes.[48, 49] This model proposes that the surface of human leukocytes is composed of “protein islands” of approximately 200-300 nm in diameter. Particular groups of proteins are segregated in separate islands. They are anchored to the cytoskeleton by protein contacts, and the lipid composition within the islands is different from the areas outside of the islands. The islands have a degree of freedom for lateral movement on the surface, and specific events can cause the relocation of one island into the immediate vicinity of another to facilitate protein interaction. This has been reported for example for the T cell antigen receptor (TCR) and its co-factor Lat.[49] On the surface of resting T cells these two proteins were found in different protein islands, which concatenated upon T cell activation. If we assume this model to be valid, and if two antigens, which are targeted by a multispecific antibody-derivative such as our triplebody, reside in separate protein islands, and if the intrinsic cross-arm binding property of the employed molecule is unsuited for simultaneous binding, and if none of the two antigens can relocate to the other island, then there is no benefit in multispecificity. However, if two different antigens can be bound simultaneously on the same cell, then cancer cell selectivity may be possible and several immunotherapeutic mechanisms of action, such as growth factor receptor inhibition, neutralization of immune evasion mechanisms, or induction of apoptosis and ADCC (or RDL), can potentially be combined.

In this proof-of-principle study, we have demonstrated that triplebodies which are bivalent, but monospecific for the target antigen, can recruit one of the most desirable effector cell populations, the cytolytic T cells, for cancer cell lysis. It remains to be investigated in future studies, whether it is possible to further enhance the value of T cell-recruiting triplebodies as potential therapeutic agents by endowing them with the dual-targeting option, based on the rational choice of a pair of target antigens on the cancer cell, which is more abundant and/or more accessible on the surface of the cancer cell than on the corresponding healthy cells. It also remains to be seen, whether the triplebodies have the improved plasma half-life relative to the BiTE agents in humans, which has been demonstrated for them in mice. However, we anticipate that both of these goals are within reach, and therefore, that triplebodies have significant therapeutic potential for the treatment of cancer and other diseases.

## METHODS

### Bacterial strains and cell lines

DNA plasmids for the eukaryotic expression of antibody-derived fusion proteins were amplified in *Escherichia coli* strain XL-1. The mammalian production cell line 293F was purchased from Life Technologies™ and cultured in serum-free FreeStyle™ medium (Life Technologies™). The pro B-ALL cell line SEM has been continuously propagated in our laboratory since its establishment in 1995,[50] the CD19-positive pre-B cell line Nalm-6, the Burkitt lymphoma cell lines Raji and Namalwa and the plasma cell leukemia cell line ARH77 were purchased from the Leibniz Institute DSMZ (German Collection of Microorganisms and Cell Cultures).[51] All cell lines were cultured in RPMI 1640, GlutaMAX™ supplemented with 10% fetal calf serum (FCS) and Penicillin (100 U/mL)/Streptomycin (100 μg/mL) (Gibco^®^, Life Technologies™). The medium for the Namalwa cell line was additionally supplemented with 1 mM sodium pyruvate.

### Construction, expression and purification of triplebody 19-3-19 and control proteins

All antibody derivatives employed in this study were cloned into the mammalian expression vector pSecTag2-HygroC (Life Technologies™). The scFv building blocks for the bispecific scFv proteins 19-3 and the Her2-3, and for the triplebodies 19-3-19 and Her2-3-Her2 were developed by our team at the University of Erlangen (CD19-specific scFv) or provided by M. Peipp (University of Schleswig-Holstein, Kiel; CD3ε- and Her2-specific scFvs), respectively. The sequences coding for the disulfide-stabilized CD16-specific scFv in the gene encoding the triplebody 19-16-19 (derived from Kellner et al. 2008, but with humanized scFv sequences) were replaced with the murine CD3ε-specific scFv sequence amplified by polymerase chain reaction (PCR) from the sequence coding for Her2-3 by standard molecular cloning methods. Similarly, the sequences coding for the Her2-specific scFv in the Her2-3 construct were replaced with the sequences coding for the humanized CD19-specific scFv to produce the coding sequences for 19-3. The sequences coding for the CD19-specific scFvs in the coding construct for triplebody 19-3-19 were replaced with coding sequences for the Her2-specific scFv amplified by PCR from the construct coding for Her2-3 to produce the coding sequences for triplebody Her2-3-Her2. (Details of the construction scheme are listed in Supplementary Table S1). 293F cells were transfected with the respective expression vectors using the *Trans*IT^®^-LT1 transfection reagent (Mirus^®^ Bio LLC) following manufacturer's instructions. Antibody-derived fusion proteins were purified by affinity chromatography from cell culture supernatants that were harvested 7 days post transfection. A Nickel-nitrilo triacetic acid (Ni-NTA) matrix was used. Protein concentrations were determined by measurement of their absorbance at 280 nm. Protein identity was confirmed and purity assessed by SDS-polyacrylamide gel electrophoresis and Western Blotting (N-terminal integrity confirmed with anti-Strep- and C-terminal integrity confirmed with anti-His antibodies).

### Preparation of peripheral blood mononuclear cells (PBMCs) from whole blood

10 - 60 mL of peripheral blood was drawn into EDTA monovettes (Sarstedt) from healthy unrelated donors and patients suffering from different types of B cell neoplasias, after informed written consent was obtained. This study is in accordance with the declaration of Helsinki and was approved by the ethics committee of the Medical Faculty of the Ludwig-Maximilians-Universität München (project no. 173-13). PBMCs were separated by density gradient centrifugation using Lymphoprep™ (Axis Shield PoC) medium, and residual erythrocytes were lysed by incubation with Ery-Lysis-Buffer (University Pharmacy, Munich) for 5 min. To generate effector cells for standard 3 hr cytotoxicity assays, an *ex vivo* expansion and stimulation of mononuclear cells (MNCs) was carried out for 20 d in the presence of IL-2 as described.[52] For T cell activation assays freshly isolated, non-stimulated PBMCs were used.

### Enrichment of a pan T cell population by preparative magnetic cell separation

Pan T cells were isolated from *ex vivo* expanded MNCs or freshly isolated, non-stimulated PBMCs by negative selection using a commercial Pan T cell isolation kit (Miltenyi Biotec, Bergisch Gladbach, Germany) according to the manufacturer's instructions. The enriched T cells are referred to as “untouched cells” because they have no residual antibody bound to their surface and have been maintained under mild buffer/medium conditions. T cell purity was assessed by flow cytometry and the purified T cells were used for binding and T cell activation studies.

### Flow cytometry

An Accuri C6 flow cytometer (BD Biosciences, Heidelberg, Germany) was used for flow cytometric analysis of the binding behavior of the antibody-derived fusion proteins and for the differential analysis of leukocyte/tumor cell subpopulations. In the Accuri C6 instrument the laser and optical alignments have been pre-set and locked. In this instrument the detector voltages are not adjustable as opposed to other machines. Equilibrium binding constants (affinity constants, K_D_) for CD19 and CD3 were determined by calibrated cytofluorimetry.[53] The maximum mean fluorescence value was set to 100% and all data points were normalized accordingly. Experiments were repeated 4 to 7 times and K_D_ values were calculated with the GraphPad Prism Software (GraphPad Software Inc., San Diego, CA, USA) using a nonlinear regression curve fit. Cell-bound antibody-derivatives were detected using a Penta-His™ AlexaFluor488-conjugated antibody (QIAGEN, Hilden, Germany). The CD3-, CD4-, CD8-, CD19-, CD25-, CD33-, CD45RA-, CD45RO-, CD56- and CD69-specific monoclonal antibodies (mAbs) used for the analysis of lymphocyte and myeloid cell content and for the detailed analysis of relevant T cell subpopulations as well as the respective isotype control mAbs were from Immunotech (Marseille, France). Specific antibody binding capacity of cells from established B-ALL lines and primary leukemia blasts for CD3 (unconjugated mAb from ebioscience, Frankfurt, Germany), CD19 (unconjugated mAb from DAKO, Hamburg, Germany), and CD33 (unconjugated mAbs from BD Pharmingen, Heidelberg, Germany) was determined with a commercial calibrated cytofluorimetric assay (QIFIKIT^®^, DAKO, Hamburg, Germany) as described.[54, 55]

### Cytotoxicity assay

To quantitate cell-mediated cytolysis (referred to as redirected lysis, RDL) induced by the BiTE or triplebody proteins, target cells were pre-labelled with Calcein AM (Life Technologies) and mixed with effector cells in RPMI 1640 GlutaMAX medium supplemented with 10% fetal calf serum at an E : T ratio of 10 : 1 unless otherwise stated. Either MNCs expanded *ex vivo* for 20 days and pre-stimulated with anti-CD3 mAb OKT3 and IL-2,[11, 56] or untouched T cells isolated via magnetic separation, were used as effector cells. After addition of different antibody-derived proteins to 200 μL reaction volume in round-bottom 96-well plates, the reactions were incubated at 37 °C with 5% CO_2_. Calcein release was determined by measuring the fluorescence intensity (relative light units, RLU) in the supernatant with a Berthold Mithras plate reader (Berthold technologies, Bad Wildbad, Germany). Maximum lysis was achieved by addition of 50 μL of a solution containing 10% Triton X-100 in RPMI 1640 GlutaMAX medium supplemented with 10% fetal calf serum and 1% Penicillin/Streptomycin. Specific lysis was calculated as follows:

% specific Lysis = 100*[RLU (sample) − RLU (background)] / [RLU (maximal lysis) − RLU (background)]

### T cell activation assay

Activation of resting T cells was determined by measurement of IFN-γ release and the expression of the early activation surface marker CD69 and the late activation marker CD25, the alpha-subunit of the high-affinity receptor for interleukin-2 (IL-2R). Freshly isolated PBMCs from healthy unrelated donors remained either untouched, were depleted of CD19-positive cells by magnetic separation, or were mixed with SEM cells at an E : T ratio of 10 : 1. Triplebody was added at a concentration of 1 nM to a 280 μL reaction mixture containing 1,4*10^5 PBMCs with or without 1.4*10^4 SEMs in RPMI 1640 + GlutaMAX™ medium with 10% FCS and 1% Pen/Strep (Gibco^®^, Life Technologies™). After incubation for 0, 24, 48 and 72 hrs the total number of living cells in the reaction mixture was determined with a hemocytometer (Marienfeld Superior, Lauda-Königshofen, Germany) using Trypan Blue staining (Gibco^®^, Life Technologies™). The surviving cells were stained for CD3/CD19, CD3/CD25, CD3/CD69 and CD3/CD45RO and the fraction of the relevant cell populations (CD19-positive targets, CD3-positive effectors, activated CD3-positive effectors and CD3 CD45RO double-positive memory T cells) was assessed by flow cytometry. The fraction of depleted B cells, i.e. the differential removed by the depletion, was computed with the help of the following formula:

% depleted B cells = 100% * (CD19^+^ cells control − CD19^+^ cells sample) / (CD19^+^ cells control)

### Measurement of IFN-γ release into peripheral blood samples

Induction of cytokine release by immune effector cells due to the presence of the triplebody or BiTE control molecule was determined in whole blood assays. Triplebodies 19-3-19 or Her2-3-Her2 or the protein 19-3 were added to 200 μL of peripheral blood in a round-bottom Nunc™ 96-Microwell plate (ThermoFisher Scientific, MA, USA) at different concentrations and were incubated for 6 hrs at 37 °C with 5% CO_2_. The samples were then diluted 1 : 1 with PBS and concentrations of IFN-γ were determined using commercial Ready-Set-Go ELISA kits (ebioscience, Frankfurt, Germany). Depending on sample availability, samples were run in duplicates or triplicates.

### Statistical analysis

All statistical analyses were performed by GraphPad Prism Software (GraphPad Software Inc., San Diego, CA, USA) using Student's t-test for the determination of significance, defined by p < 0.05.

## SUPPLEMENTARY TABLE



## ABBREVIATIONS

ADCC: antibody-dependent cellular cytotoxicity
ALL: acute lymphoblastic leukemia
B-CLL: chronic B cell leukemia
BiTE: bispecific T cell engager
bsscFv: bispecific single chain Fv
CD: cluster of differentiation
CI: confidence interval
CR: complete remission
EC50: concentration at which half maximal effect is achieved
ELISA: enzyme-linked immunosorbent assay
FACS: fluorescence-assisted cell sorting
IFN-γ: Interferon gamma
IL-2: Interleukin-2
MFI: Mean Fluorescence Intensity
MNC: mononuclear cells
MPAL (NOS): mixed phenotype acute leukemia (not otherwise specified)
NHL: Non-Hodgkin Lymphoma
PB: peripheral blood
PBMC: peripheral blood mononuclear cells
PCR: polymerase chain reaction
PHA: Phytohemagglutinin
pI: isoelectric point
pre-B cells: μ-positive, CD10 (CALLA)-positive
pro-B cells: μ-negative, CD10 (CALLA)-negative
RDL: redirected lysis assay
scFv: single chain fragment variable
SEM: standard error of the mean
TAA: tumor-associated antigen
TNFα: tumor necrosis factor alpha.
