# Supplementary material for: T cell-recruiting triplebody 19-3-19 mediates serial lysis of malignant B-lymphoid cells by a single T cell
Source: Oncotarget. 2014 Jul 23;5(15):6466–83. doi: 10.18632/oncotarget.2238 (PMC4171644; doi:10.18632/oncotarget.2238)
Supplement: Supplementary file 1 [file oncotarget-05-6466-s001.pdf]

SUPPLEMENTARY TABLE

Supplementary Table S1. Computed protein characteristics of the T cell-engaging bispecific scFv molecules and triplebodies. Protein parameters calculated from the primary sequences of the engineered T cell-engaging BiTEs and triplebodies with the ProtParam (http://web.expasy.org/protparam/) and PeptideCutter (http://web.expasy.org/peptide_cutter/) tools[41].

Protein	Length [aa]	MW [kDa]	ε [M^{-1} cm^{-1}]	computed pI	Construct	Relevant Proteinases
19-3	572	60.747	117,620	8.52	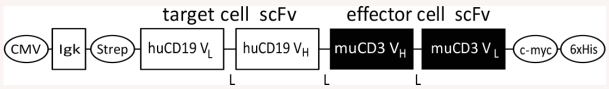	Chymotrypsin, Clostripain, Pepsin, *Proline endopeptidase (4 sites)*, Trypsin
19-3-19	854	89.94	168,180	8.53	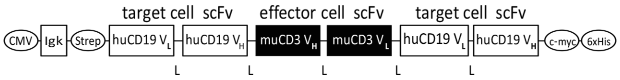	Chymotrypsin, Clostripain, Pepsin, *Proline endopeptidase (5 sites)*, Trypsin
Her2-3	563	59.503	117,160	8.66	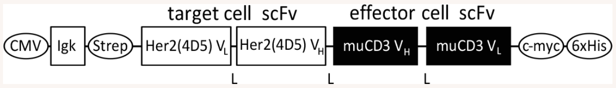	Chymotrypsin, Clostripain, Pepsin, *Proline endopeptidase (4 sites)*, Trypsin
Her2-3-Her2	834	87.249	167,260	8.71	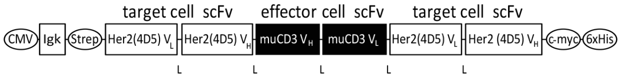	Chymotrypsin, Clostripain, Pepsin, *Proline endopeptidase (5 sites)*, Trypsin
